# Buffalo Cheese Whey Proteins, Identification of a 24 kDa Protein and Characterization of Their Hydrolysates: *In Vitro* Gastrointestinal Digestion

**DOI:** 10.1371/journal.pone.0139550

**Published:** 2015-10-14

**Authors:** Juliana C. Bassan, Antonio J. Goulart, Ana L. M. Nasser, Thaís M. S. Bezerra, Saulo S. Garrido, Cynthia B. Rustiguel, Luis H. S. Guimarães, Rubens Monti

**Affiliations:** 1 Faculdade de Ciências Farmacêuticas, UNESP Univ EstadualPaulista, Departamento de Alimentos e Nutrição, Araraquara - SP, Brazil.; 2 Instituto de Química, UNESP Univ EstadualPaulista, Departamento de Bioquímica e Química Tecnológica, Araraquara - SP, Brazil.; 3 Universidade de São Paulo, Departamento de Biologia, Ribeirão Preto - SP, Brazil; CNR, ITALY

## Abstract

Milk whey proteins are well known for their high biological value and versatile functional properties, characteristics that allow its wide use in the food and pharmaceutical industries. In this work, a 24 kDa protein from buffalo cheese whey was analyzed by mass spectrometry and presented homology with *Bos taurus* beta-lactoglobulin. In addition, the proteins present in buffalo cheese whey were hydrolyzed with pepsin and with different combinations of trypsin, chymotrypsin and carboxypeptidase-A. When the TNBS method was used the obtained hydrolysates presented DH of 55 and 62% for H1 and H2, respectively. Otherwise for the OPA method the DH was 27 and 43% for H1 and H2, respectively. The total antioxidant activities of the H1 and H2 samples with and without previous enzymatic hydrolysis, determined by DPPH using diphenyl-*p*-picrylhydrazyl radical, was 4.9 and 12 mM of Trolox equivalents (TE) for H2 and H2Dint, respectively. The increased concentrations for H1 and H2 samples were approximately 99% and 75%, respectively. The *in vitro* gastrointestinal digestion efficiency for the samples that were first hydrolyzed was higher compared with samples not submitted to previous hydrolysis. After *in vitro* gastrointestinal digestion, several amino acids were released in higher concentrations, and most of which were essential amino acids. These results suggest that buffalo cheese whey is a better source of bioavailable amino acids than bovine cheese whey.

## Introduction

Cheese whey, which is a byproduct of cheese production, is a greenish-yellow solution composed of water, lactose, proteins and minerals [1–2] that represents 85–90% of the milk volume [3]. Dairy experts from around the world have sought to use whey because it contains highly nutritious components that should not be wasted and because it is a potent environmental pollutant [4]. Milk whey proteins are well known for their high biological value and versatile functional properties, characteristics that allow its wide use in the food [5] and pharmaceutical [6] industries. These compact and globular proteins are responsible for 20% of the total protein contained in milk and, unlike casein, remain soluble at pH 4.6 [7]. The main proteins present in cheese whey are β-lactoglobulin (3.2 g.L^-1^, 18.3 kDa), α-lactalbumin (1.2 g.L^-1^, 14.2 kDa), serum albumin (0.4 g.L^-1^, 66.0 kDa), immunoglobulin (0.8 g.L^-1^, 146–1030 kDa), and lactoferrin (0.2 g. L^-1^, 80 kDa), among others [2,8]. Current technologies allow the separation, isolation and purification of proteins from whey, usually through a combination of methods, such as filtration techniques associated with chromatography [5]. A wide variety of products, such as concentrates (35–80% protein), protein isolates (minimum protein content of 90%), protein fractions (α-lactalbumin, β-lactoglobulin and lactoferrin) and hydrolysates [4], which are classified as GRAS for use in food products [9,4], are available on the market. The physical, chemical and functional properties of macromolecules such as proteins can be modified and improved by enzymatic hydrolysis, particularly the absorption capacity, without affecting their nutritional value [10], while reducing allergenic processes [11]. Protein hydrolysates of cheese whey are a source of bioactive peptides [12] with opioid, antihypertensive, antithrombotic, antioxidant, immunomodulatory and antimicrobial activities [13–17] and of essential amino acids [18–19], which are often used as protein supplementation for infants and athletes and for parenteral administration [20]. There are few reports in the literature concerning bioactive peptides obtained by the hydrolysis of whey proteins, suggesting that further studies are required to fully understand this byproduct [21].

Genetic variants have being identified in buffaloes that are present in different fractions of milk proteins, affecting the composition and technological properties of these fractions [22]. In addition, studies performed in different parts of the world show that different buffalo breeds present both variants of α-lactalbumin and β-lactoglobulin [23–24]. Although bovine cheese whey proteins have been extensively studied, studies regarding buffalo milk proteins are quite scarce [25].

In this article, we present a methodology to hydrolyze the proteins from Murrah buffalo cheese whey, and a study of the hydrolysis products. We also describe the amino acid sequencing of a discovered variant protein of β-lactoglobulin.

## Materials and Methods

In all cases, the experiments were performed in triplicate, and the experimental error was never greater than 5%.

### Reagents

Pepsin, trypsin, chymotrypsin, carboxypeptidase-A, bile salts, pancreatin, (±)-6-hydroxy-2,5,7,8-tetramethylchromane-2-carboxylic acid (Trolox) and 2,2 diphenyl-1-picrylhydrazyl (DPPH) were purchased from Sigma-Aldrich Co. Renin from *Aspergillus niger* var. *awamori* (Chr. Hansen Ind. Com. Ltd) was acquired in the local market. Other reagents were of analytical grade.

### The productin of buffalo cheese whey

The buffalo cheese whey was prepared by adding 0.6% (v/v) renin from *A*. *niger* and 0.5% (v/v) 0.5 mol. L^-1^ CaCl_2_ to buffalo milk at 35°C for 1 h. After casein precipitation, the whey was filtered in gauze [26] and dialyzed in cellulose membrane (12 kDa, Sigma) under constant magnetic stirring at 8°C. Periodic water exchange for lactose removal was also performed [27]. Fat was removed by adsorption in kaolin (20 g. L^-1^ w/v), with subsequent centrifugation at 7400 x *g* at 4°C for 30 min. The treated whey was stored at -18°C until further use.

### Total protein, lactose and fat determination

The concentrations of protein, lactose and fat were determined following the methodologies of Bradford [28], Miller [29] and Gerber [30], respectively. Absorptivities were 0.0208 (μg prot. mL^-1^) ^-1^. cm^-1^ and 247.23 mol. L^-1^. cm^-1^, using bovine serum albumin (BSA) and lactose as standards, respectively.

### PAGE and SDS-PAGE analysis of buffalo chees whey proteins

Ten percent PAGE [31] and 12% SDS-PAGE [32] analyses were performed to obtain the whey proteins profiles and to monitor and analyze the hydrolysis of these proteins. Protein bands from PAGE and from SDS-PAGE were stained with silver [33] and with Brilliant Blue G-Colloidal (Sigma), respectively. For the SDS-PAGE analysis, a GE Healthcare Life Sciences molar mass standard composed of phosphorylase b (97 kDa), bovine serum albumin (66 kDa), ovalbumin (45 kDa), carbonic anhydrase (30 kDa), trypsin inhibitor (20.1 kDa) and α-lactalbumin (14.4 kDa) was used.

### Molar mass determination of the variant protein from the buffalo cheese whey

**Fig 1:**
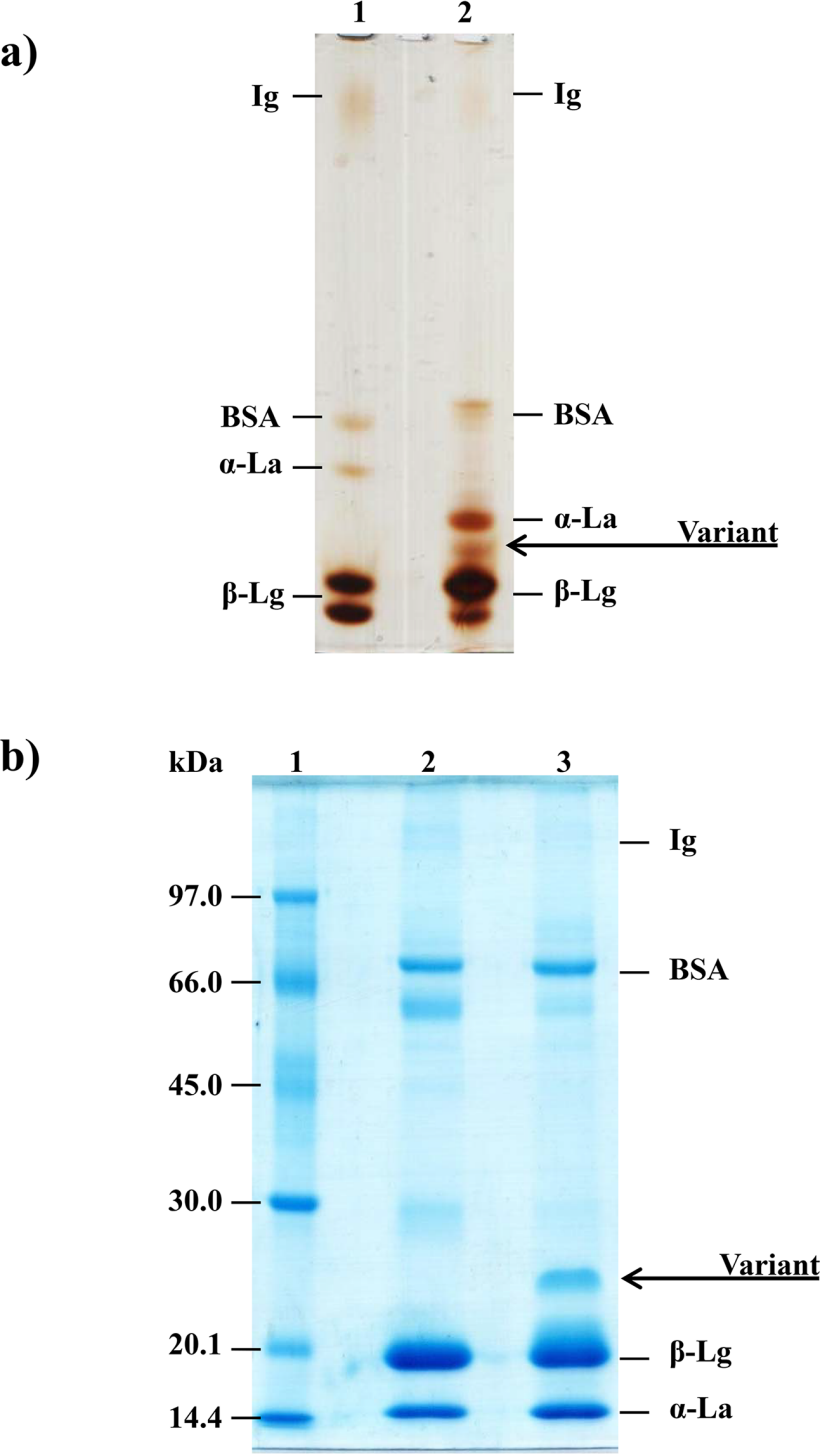
Electrophoretic profile of the buffalo cheese whey. a) Ten percent PAGE silver stained protein profile of treated bovine (1) and buffalo (2) whey. b) Twelve percent SDS-PAGE of the bovine and buffalo milk whey proteins. (1) Molar mass standards; (2) bovine milk whey; (3) buffalo milk whey. Ig: immunoglobulin; BSA: bovine serum albumin; α-La: alpha-lactalbumin; β-Lg: beta-lactoglobulin.

The molar mass of the new protein was determined from the SDS-PAGE (Fig 1A and 1B) using the Least Square Method, according to the formula:

$$\left[\begin{array}{cc} \sum_{k=1}^{n}{x_{i}}^{2} & \sum_{k=1}^{n}x_{i}\\ \sum_{k=1}^{n}x_{i} & n \end{array}\right]\times \left[\begin{array}{c} a_{1}\\ a_{2} \end{array}\right]=\left[\begin{array}{c} \sum_{k=1}^{n}f\left(x_{i}\right)x_{i}\\ \sum_{k=1}^{n}f\left(x_{i}\right) \end{array}\right]$$

Where:

*x*_*i*_: Rf value

*f*(*x*_*i*_): The Molar Mass log

*a*_1_: Constant to be found

*a*_2_: Constant to be found

*n*: Number of elements (proteins).

Cited constants above are the function constants *g*(*x*) = *a*_1_ *x* + *a*_2_, there is, the function modeling the experiment.

### Isolation and characterization of the 24 kDa protein from the buffalo cheese whey

Proteins were 10-fold concentrated by lyophilization and submitted to 12% SDS-PAGE as previously described. After the running, the gel was processed to visualize the proteins of interest and to serve as a guide for cutting slices containing the 24 kDa protein.

The gel slices were dried and submitted to digestion using 0.5 μg of trypsin (Promega) in 0.1 M sodium bicarbonate buffer, pH 8.0. After digestion, the peptides were loaded in a Poros 50 R2 reverse phase column (Perseptive Biosystems). The purified peptides were added with matrix solution (50 mg. mL^-1^ α-ciano-4-hidroxycinnamic acid, 50% acetonitrile and 0.1% trifluoroacetic acid), and 2 μL samples were used for MALDI-TOF/TOF-MS analysis. The MS/MS profiles were analyzed using the MASCOT software (Matrix Science, London, UK) and the Swiss Prot and NCBInr databases.

### Enzymatic hydrolysis of cheese whey proteins

After removing the fat and lactose, the whey (10 mL) was hydrolyzed with pepsin (429 U, pH 3.0; 50°C), trypsin (432 U), chymotrypsin (18.3 U) and carboxypeptidase-A (16.7 U). Trypsin, chymotrypsin and carboxypeptidase-A were added to the whey at 50°C and pH 9.5 by two different methods (M1 and M2). In the M1, the enzymes were simultaneously added, and the time of hydrolysis was 180 min, producing the H1 hydrolysate, whereas in the M2, these same enzymes were added to the whey with a 10 min interval between each addition, at the same pH and temperature, with a 24 hours reaction, producing the H2 hydrolysate. The end of the hydrolysis was determined by SDS-PAGE and by HPLC analysis, performed with samples collected at different reaction times and that were incubated in a boiling bath to denature the enzymes used.

### Antioxidant activity of the buffalo cheese whey hydrolysates

Briefly, 1 mL mmol. L^-1^ DPPH^•^ solution in methanol 80% was mixed with 0.5 mL of samples and standard. The mixture was kept at room temperature for 30 min; then the absorbance at 517 nm was measured in a BioTek Synergy H1 reader (BioTek Instruments, USA). Trolox (0–150 μmol. L^-1^) was used as standard. The results were expressed as a Trolox Equivalent (TE) per μmol. L^-1^ [34].

### Quantification of the degree of hydrolysis (DH) using OPA and TNBS

The OPA assay was carried out with a modification of the Spellman et al. method [35], adding 0.02 mL of sample or standard to 2.4 mL of OPA/NAC reagent without SDS (the modification of the method). The OPA/NAC reagent was prepared mixing 10 mL OPA (50 mmol. L^-1^), 10 mL NAC (50 mmol. L^-1^) and 80 mL borate buffer (0.1 mol. L^-1^) pH 9.5. After 10 min the absorbance of this solution was measured at 340 nm. A standard curve was prepared using *L*-isoleucine (0–2 mg. mL^-1^). DH values were calculated using Eqs 1 and 2.

$$DH\;\%=\frac{100n}{N}$$ (1)

$$n=\frac{\Delta ABS\times M\times d}{\varepsilon \times P}$$ (2)

Where N is the the total number of peptide bonds per protein molecule and *n* is the average number of peptide bonds hydrolyzed. The ΔABS is the difference in the absorbance of the hydrolyzed and unhydrolized whey. The component d is the dilution factor, ε is the molar extinction coefficient (mol. L^-1^. cm^-1^) and P the protein concentration of the sample (g. L^-1^). The average molecular mass of proteins in the whey (M) was 26.6 kDa and the peptide bonds per protein molecule (N) was 204.

For the TNBS assay the method used was modified from Spadaro et al. [36]. In this assay 0.2 mL of sample or standard was added to 0.4 mL borate-NaOH buffer (5 mmol. L^-1^) pH 9.5 and 0.4 mL TNBS (5 mmol. L^-1^). The mixture was incubated at room temperature for 40 min. The reaction was stopped by the addition of 0.2 mL 18 mmol. L^-1^ Na_2_SO_3_ prepared in 2 mol. L^-1^ NaH_2_PO_4_ and the absorbance was measured at 420 nm. *L*-Leucine (0.02–1.5 mmol. L^-1^) was used as standard and the hydrolysis was calculated using the following formula (Eq 3):

$$DH\;(\%)=100\frac{\left({AN}_{2}-{AN}_{1}\right)}{Npb}$$ (3)

Where AN_1_ is the amino nitrogen content of the protein substrate before hydrolysis (mg. g^-1^) and AN_2_ is the amino nitrogen content of the protein substrate after hydrolysis (mg. g^-1^). The nitrogen content of the peptide bonds in the whey (Npb) was 123.3 mg. g^-1^ [37]. The values of AN were obtained using the following formula (Eq 4):

$$AN=\frac{ABS1}{\left(\varepsilon \times P\right)}$$ (4)

Where ABS is the absorbance of the sample, ε is the molar extinction coefficient for *L*-Leucine (mg. L^-1^) and P is the protein concentration of the sample (g. L^-1^).

### Simulation of the *in vitro* gastric and intestinal enzymatic digestion (determination of the dialyzability)

The simulation of gastric and intestinal enzymatic digestion was performed according to Luten et al. [38]. In this procedure, 95 mL of integral proteins (non-hydrolyzed whey-NH) and hydrolysates H1 and H2 were adjusted to pH 2.0 with 6 mol. L^-1^ HCl, followed by the addition of 2 mL of Milli-Q water and 3 mL of pepsin (1.98x10^5^ U in 0.1 mol. L^-1^ HCl) at 37°C for 2 h, simulating gastric digestion. Then the mixture was placed at 4°C for 10 min to minimize pepsin activity. The peptic digest sample was separated into three aliquots (20 mL each); the aliquots were transferred to beakers containing cellulose membranes filled with 0.5 mol. L^-1^ NaHCO_3_ at 37°C for the dialysis of molecules with molar masses smaller than 12.4 kDa. When pH 7.0 was reached, 5 mL of 0.1 mol. L^-1^ NaHCO_3_ containing 20 mg of pancreatin + 125 mg of bile salts were added, and the mixture was kept in contact with the membrane for 2 h, simulating intestinal digestion. For determination of the amount of NaHCO_3_ required to simulate intestinal pH two aliquots (20 mL each) of the peptic digest were separated to determine the titratable acidity using 0.5 mol. L^-1^ NaOH until reaching pH 7.5 and 0.5 mol. L^-1^ of gram-equivalent.

### Peptides and amino acids from the hydrolysis products and enzymatic digest analyses

H1 and H2 hydrolysates and the dialysates from the dialyzability experiments (solutions inside the membrane) were filtered using a GV Millex 0.45 μm unit (Millipore) and analyzed by HPLC (Varian ProStar or Shimadzu LC 10A), which was equipped with a reverse phase column Nucleosil C18 (25 x 0.46 inch, 5 μm particle size, 300 Å pore size). The eluents of the mobile phase were as follows: A) water and 0.045% trifluoracetic acid (TFA) and B) acetonitrile (ACN) and 0.036% TFA, with a 5–95% linear gradient of eluent B, a flow rate of 1.0 mL. min^-1^ for 30 min and with UV detection at 220 nm.

Analysis of the released amino acids was performed using a LC-10A/C-47A Shimadzu automatic analyzer with a fluorescence detector, which was equipped with a Shimadzu Shim-pack ion-exchange column with *o*-phtalaldehyde (OPA) post-column functionalization of the amino acids. Mobile phase A consisted of 19.6 g sodium citrate, 140 mL ethanol 99.5%, 16.7 mL perchloric acid 60%, with a final 1 L volume at pH 10; mobile phase B was 58.8 g sodium citrate, 12.4 g boric acid, 30 mL 4.0 mol. L^-1^ sodium hydroxide solution, with a final 1 L volume at pH 10; mobile phase C consisted of 0.2 mol. L^-1^ sodium hydroxide solution at pH 13.5, with a flow rate of 0.6 mL. min^-1^. A standard amino acid mixture was used to calibrate the system and to provide the elution time of each amino acid and the conversion factor between the peak area and the concentration of each sample. The molar ratio of amino acids was established, assuming the concentration of the amino acid closest to the mean for all residues as one unit.

## Results and Discussion

### Protein, lactose and fat levels in buffalo cheese whey

The protein concentrations were obtained after dialysis, which removed interfering components, such as oligopeptides and amino acid residues released during the enzymatic coagulation of milk. Lactose and fat concentrations decreased by 98.5% and by more than 90%, respectively, after dialysis and filtration with kaolin (Table 1). The removal of these compounds is important for determination of protein concentration with as little as possible interfering.

**Table 1:** Protein, lactose and fat concentrations of the *in natura* and treated buffalo milk whey.

Conditions	Protein (g. L^-1^)	Lactose (g. L^1^)	Fat (%)
Integral *in natura* whey	11.13±0.13	65.30±2.20	0.90±0.17
Dialyzed whey*	7.33±0.15	1.23±0.14	0.60±0.10
Treated whey**	6.53±0.49	1.00±0.14	< 0.10
Bovine milk whey (Romám, et al. 2011)	5.40	42.60	2.00

*reduction of lactose

**dialyzed, defatted and centrifuged

Results reported by Romám et al. [39] for bovine cheese whey showed lower protein and lactose concentrations compared with buffalo cheese whey, with the exception of the fat concentration. These different protein, lactose and fat levels are most likely related to the analytical methods used and to the fact that the samples are from different species of mammals and, therefore, have different environmental and genetic factors that are involved, as described in the literature [40–41]. The results show that buffalo whey can be considered an excellent source of high biological value protein that can be used in the food industry.

### Comparison between cheese whey proteins from buffalo and bovine by PAGE and by SDS-PAGE

Ten percent PAGE of bovine and buffalo cheese whey proteins shows a slight difference in the migration of the protein bands equivalent to bovine serum albumin (BSA) and α-lactalbumin (α-La), as shown in Fig 1A. The PAGE also shows an extra protein (variant) band in the buffalo cheese whey in the region between the α-La and β-Lg fractions. There are few published data concerning the protein profile of buffalo cheese whey; however, a recent study performed by Buffonni et al. [25] using RP-HPLC showed the presence of two protein peaks corresponding to α-La. In another study, Chianese et al. [23] used isoelectric focusing showed that α-La has two genetic variants (α-La A and α-La B).

**Fig 2:**
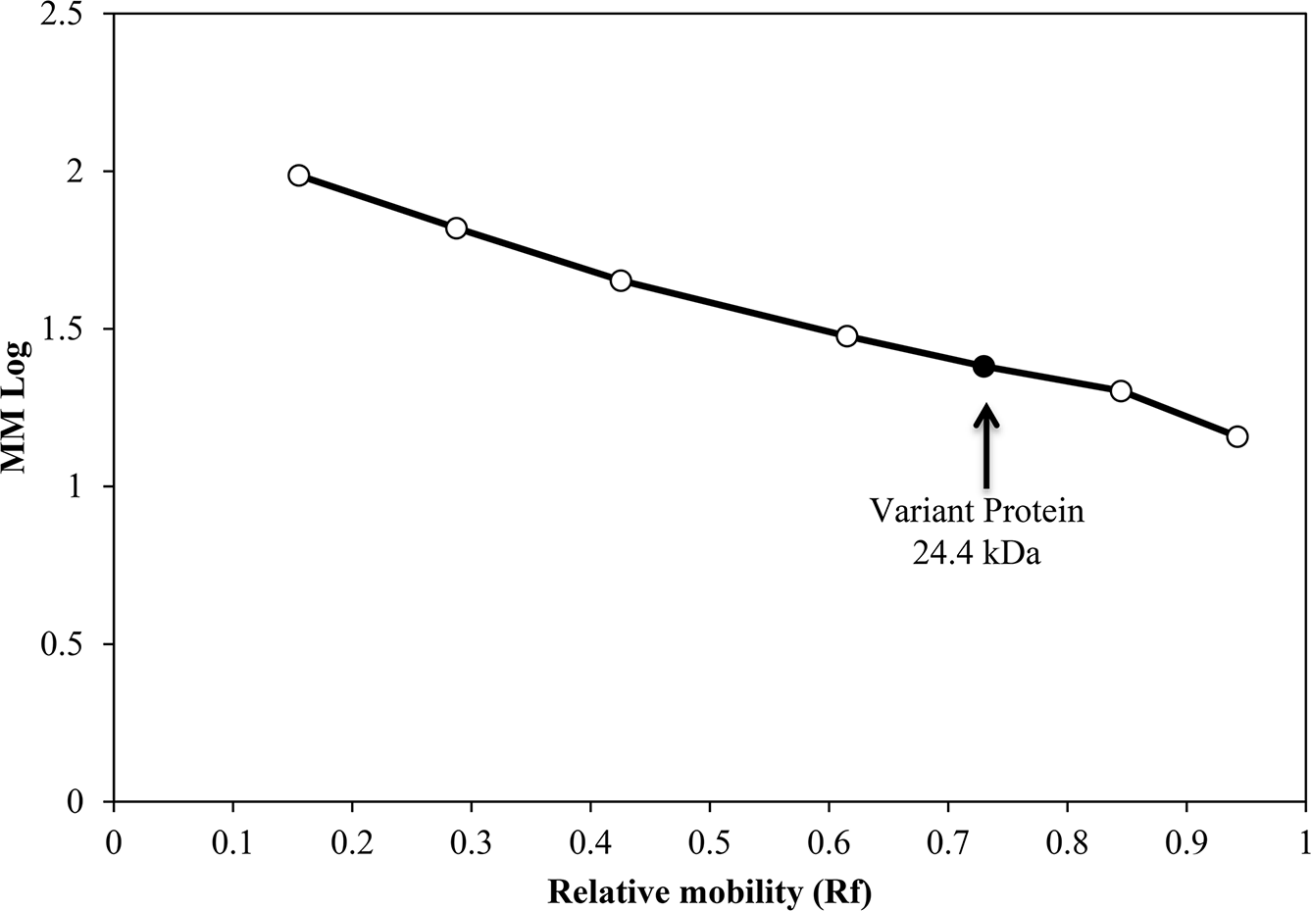
Determination of the variant protein molecular weight by Least Square Method. Calibration curve for the determination of the toxin molecular weight by SDS-PAGE (12%). Marker proteins used for calibration were phosphorylase b (97 kDa), bovine serum albumin (66 kDa), ovalbumin (45 kDa), carbonic anhydrase (30 kDa), trypsin inhibitor (20.1 kDa) and α-lactalbumin (14.4 kDa). (●) calibration curve; (○) variant protein.

Twelve percent SDS-PAGE confirmed the presence of a protein band in buffalo whey with molecular weight ranging from 20.1 to 30.0 kDa and with relative electrophoretic mobility (Rf) of 0.73 (Fig 1B) that is absent in bovine whey. The determined molar mass of the variant protein was confirmed to be 24kDa by the Least Square Method, determining the molar mass logarithm using the protein Rf (0.73) and, subsequently, the molar mass (Fig 2).

Regarding other proteins, cheese whey from buffalo milk is highly similar to bovine cheese whey, having proteins with molecular weights between 97 and 66 kDa (immunoglobulins and serum albumin, respectively) and between 20.1 and 14.4 kDa (β-lactoglobulin and α-lactalbumin, respectively). A protein with molecular mass of approximately 30 kDa is also present in both species; however, this protein is most obvious in bovine whey.

### Mass spectrometry analysis

**Fig 3:**
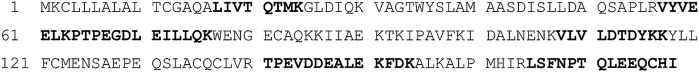
Amino acid sequence for β-Lg from *Bos taurus* (gi/229460). The tryptic peptides obtained for 24 kDa protein from buffalo cheese way were identical to the red sequence. It was obtained 37% of coverage.

Our results indicate the presence of a variant protein in the buffalo cheese whey, which can be different in the various breeds. According to the MS/MS profiles, five tryptic peptides were obtained from the 24 kDa protein. These peptides were aligned with the β-Lg sequence from *Bos taurus* with 37% of total coverage and high scores (Fig 3). On the other hand, another peptide (VGINYWLAHK) with 7% of coverage for α-La was also obtained with a reduced score. The peptide data associated to the difference found in the molecular masses (SDS-PAGE and data bank) indicates the existence of a variant β-Lg in the buffalo cheese whey. Similarly, Chianese et al. [23] found two variants for 0078-La (α-La A and α-La B). These observations reinforce the necessity of new studies concerning this field to clarify the function and importance of these new proteins.

### Determination of the DH for buffalo cheese whey hydrolysates

**Fig 4:**
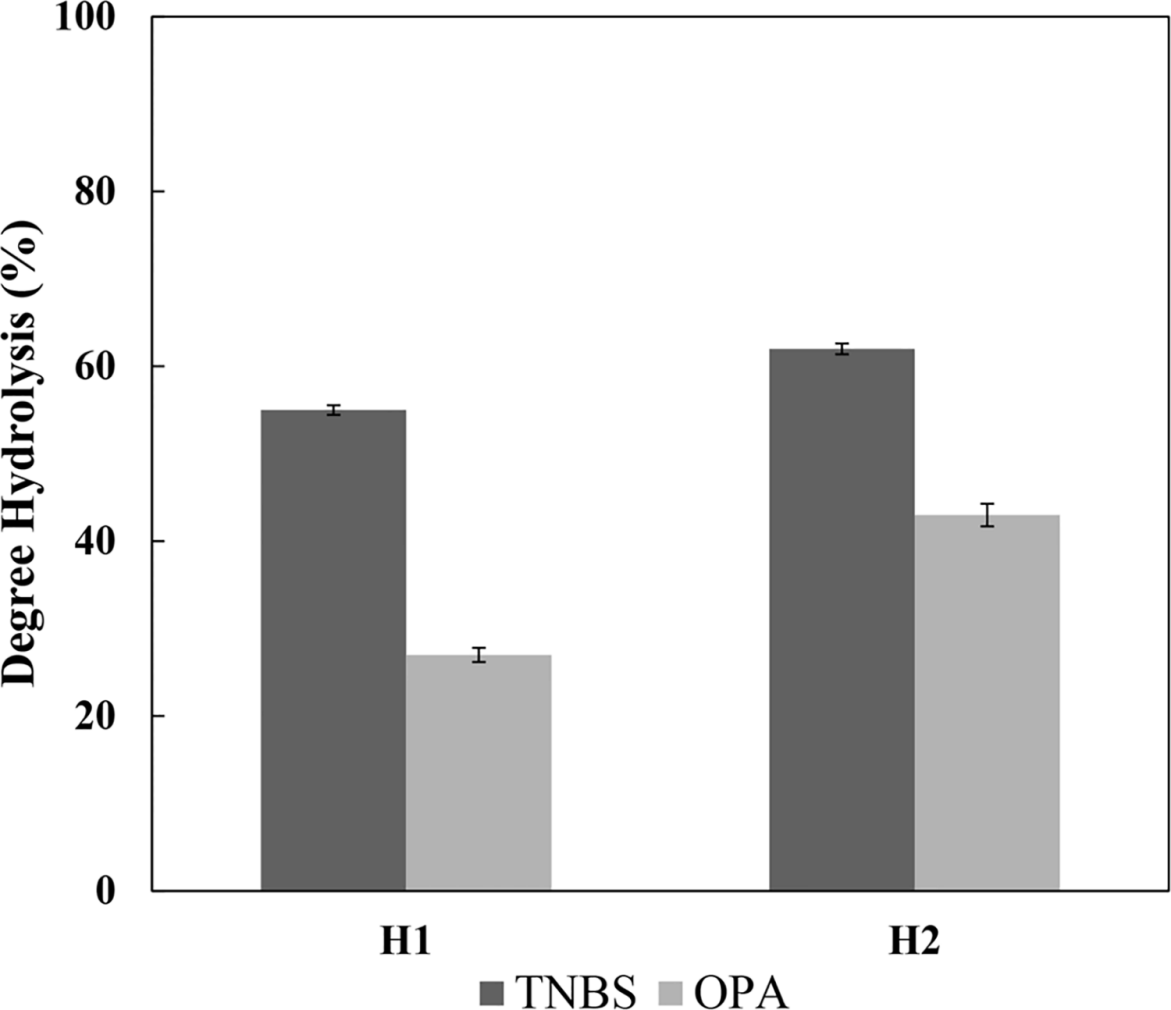
Determination of the hydrolysis degree for buffalo cheese whey hydrolysates. The values of DH were obtained by TNBS and OPA methods for hydrolysis using the M1 and M2 methods.

The TNBS reacts specifically with primary amino groups to form a trinitrophenyl (TNP) derivative that can be quantified using a colorimetric method [42]. However, this method is frequently used to quantify the extension of reactions between reducing sugars and free amino groups of proteins (Maillard reaction), where a condensation takes place involving mainly the lysine ε-NH_2_ and also the terminal α-NH_2_ [43]. In this sense, the reaction between TNBS and lysine ε-NH_2_ is a wide known process, responsible for a background that increases the values of the DH. A comparative study of the TNP-derivatives absorbance was carry out by Sashidhar and collaborators [44], where the derivative TNP-lysine had twice the absorbance of the TNP-glutamic acid, showing that TNBS reacts equally with both α-amino and ε-amino groups of amino acids. This effect was observed in determination of the DH for the buffalo cheese whey hydrolysates (Fig 4), where the values obtained with TNBS assay were higher by a factor of 2 and 1.44, for H1 and H2 respectively.

Another method is based on the specific reaction between OPA and primary amino groups in the presence of a thiol, forming isoindoles 1-alkylthio-2-alkyl-substituted that can be quantified at 340 nm [35]. The N-acetyl-*L*-cysteine can be used as thiol in this assay; however, a side reaction between NAC and cysteine residues can underestimate the DH in whey protein hydrolysates because the cysteine residues compete with NAC for position 1 in the isoindole. Once this cysteine residue is linked its quantification is not possible, since the peptides and amino acids of the sample should interact with OPA using the primary amino groups instead of thiol groups [45]. The determination of the DH for buffalo cheese whey hydrolysates using OPA presented lower values comparing with TNBS, being 27 and 43% for H1 and H2, respectively.

### SDS-PAGE and HPLC of hydrolysis products

**Fig 5:**
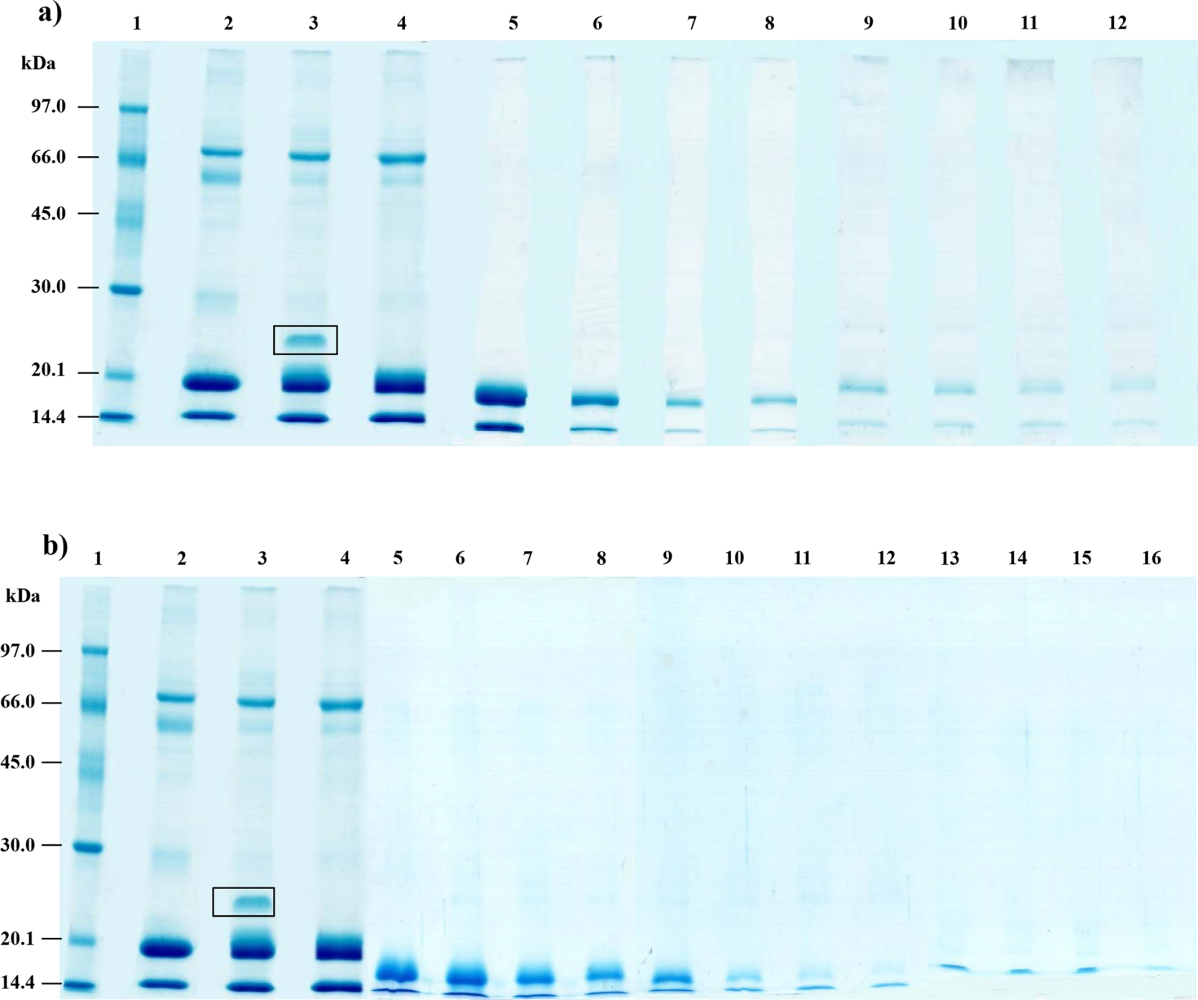
SDS-PAGE patterns for the H1 and H2 hydrolysates. In the both figures the Lane 1: molecular mass standards; Lane 2: treated bovine milk whey; Lane 3: treated buffalo milk whey. The lanes 4–12 are showing hydrolysates produced using the M1 method (Fig 5a) with incubation between 0–180 min, whereas the lanes 4–16 are showing hydrolysates produced using the M2 method (Fig 5b) with incubation between 0–1440 min.

The profile of H1 at different reaction times is showed in Fig 5A. The protein profiles of bovine and buffalo non-hydrolyzed cheese whey proteins (lanes 2–3), were used to compare the hydrolysis development by the enzymes pepsin, trypsin, chymotrypsin and carboxypeptidase-A. The disappearance of the variant protein band present in the buffalo cheese whey (lanes 4–5), and the albumin (buffalo whey), indicates its hydrolysis by pepsin during the first 20 min of the reaction at pH 3.0 and at 50°C. A gradual decrease in the color intensity of the protein bands clearly indicates the efficiency of the hydrolysis by M1 method (lanes 6 to 12). These results also show the resistance of α-La and β-Lg to hydrolysis after 180 min under the chosen experimental conditions (pH 9.5 and 50°C). The SDS-PAGE of the sample with a high degree of hydrolysis (H2) at different reaction times is shown in Fig 5B. Lanes 1 to 4 are the same as in Fig 5A (same hydrolysis conditions). When using the M2 method, we also observed that the bulk of proteins in the buffalo cheese whey were hydrolyzed after 24 hours of reaction (lanes 9–16), whereas α-La and β-Lg showed some resistance to the hydrolysis (lanes 5–8).

**Fig 6:**
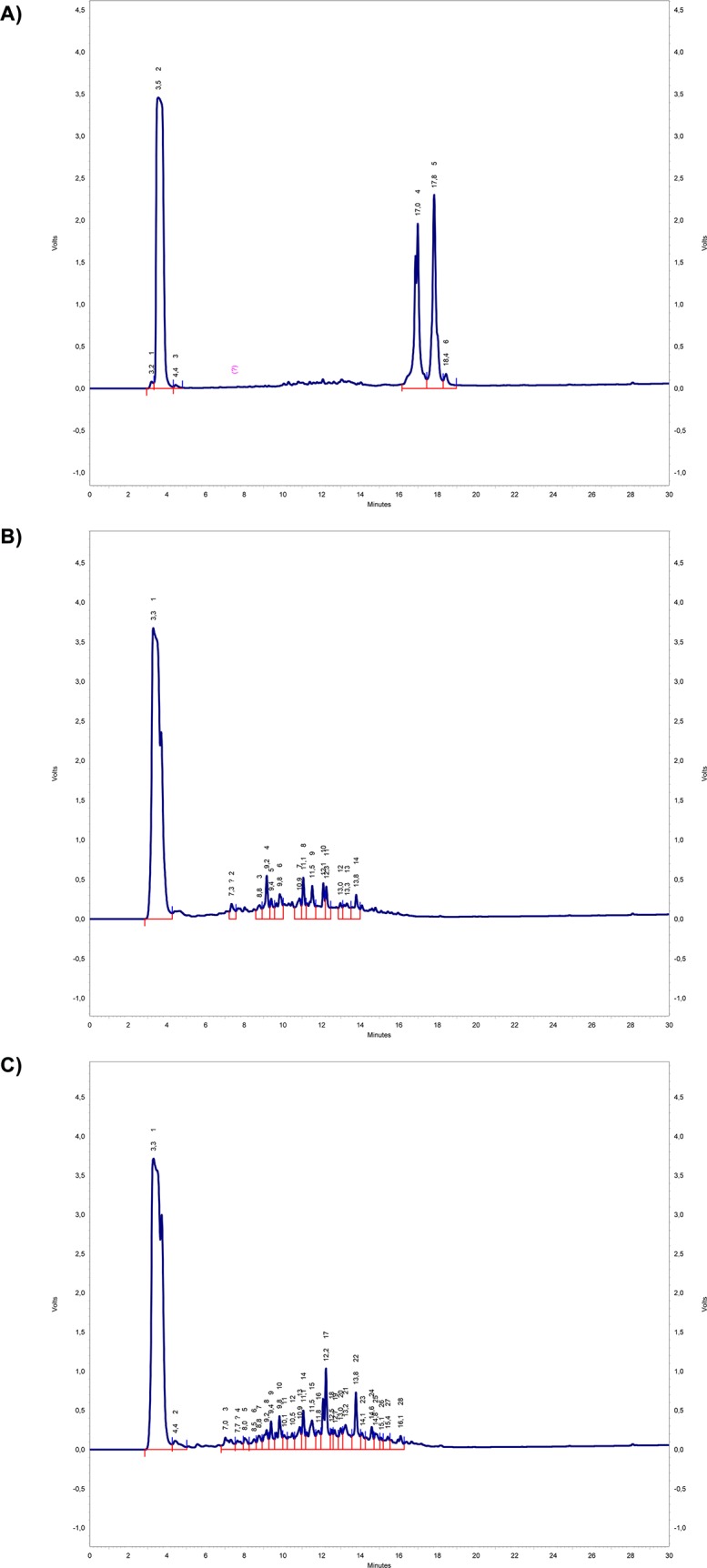
The HPLC chromatographic of the buffalo cheese whey hydrolysates. a) non-hydrolyzed (NH), b) with a medium degree of hydrolysis (H1), and c) with a high degree of hydrolysis (H2). Reverse phase chromatography using column Kromasil C18 (250 x 4.6 mm) Φ = 5 μm, 300 Ǻ porosity with a 5–95% linear gradient (solvent A: water with 0.045% TFA and solvent B: acetonitrile containing 0.036% TFA, 30 min), a flow rate of 1.0 mL min^-1^ and with detection at 220 nm.

The non-hydrolyzed (NH) buffalo cheese whey proteins were analyzed by HPLC (Fig 6A), presenting few peaks (t_R_ 17.0–18.4 min) as expected, because the proteins present in the buffalo whey were intact in relation to their polypeptide chains. These peaks were reduced with the enzymatic hydrolysis, and the number of peaks considerably increased after 180 min of hydrolysis for M1 method (Fig 6B), indicating the release of peptides (t_R_ 7.0–16.1 min); however, the hydrolysis using the M2 method (Fig 6C) was more efficient, because some peptides detected disappeared after 24 hours of reaction, increasing the release of amino acids. The HPLC results for the final hydrolysis products of M1 and M2 confirmed the SDS-PAGE results.

### Antioxidant capacity

Nowadays, the antioxidant activity of natural products has been studied, among them compounds originating from fruits and vegetables. It has also been known that peptides released during the enzymatic hydrolysis of proteins are able to modulate certain specific biological functions such as anti-hypertensive activity, opioids, antimicrobial and antioxidant. Cheese whey proteins and their hydrolysates (peptides) are potential sources of compounds with antioxidant activity [46–48].

Enzymatic digestion of cheese whey proteins enables the disruption of tertiary structure of the proteins making the most exposed and accessible amino acid residues to react with free radicals [49]. It is known that cysteine (Cys) is an amino acid that has strong antioxidant activity, being able to donate hydrogen of its thiol group [50]. Furthermore, Cys is essential for the synthesis of glutathione, an important intracellular antioxidant [18]. However, other amino acid residues as Tyr, Trp, Met, Phe, His, Ile, Leu and Pro are also able to scavenge free radicals and act as antioxidants [47,51].

In this study hydrolysates prepared using M2 method showed antioxidant activity for the both H2 (first hydrolysis) and H2Dint (after gastric and intestinal digestion—dialyzability). These samples showed 4.9 and 12 mmol. L^-1^ Trolox equivalents, respectively, being able to sequester DPPH^•^.

When a higher degree of hydrolysis was achieved, as shown in Table 2, there was a release of amino acids such as Met, Leu, Phe, His, Pro, Tyr, that have the capacity to scavenge free radicals, as previously reported in other studies [47,51].

**Table 2:** Relative concentration (nmol. L^-1^) of the amino acids from buffalo milk whey before and after dialyzability.

	Hydrolysis 1^1^			Dialyzability											
				NH				H1				H2			
Amino acid	NH	H1	H2	GD	DExt.	DInt.	Σ AA^2^	GD	DExt.	DInt.	Σ AA^2^	GD	DExt.	DInt.	Σ AA^2^
**Thr** ^**3**^	0	0	0	50.561	0	389.537	389.537	386.135	1298.012	423.932	1721.944	426.081	1134.739	398.165	1532.904
**Val** ^**3**^	0	0	0	32.910	1128.851	240.016	1368.867	228.764	685.277	216.818	902.095	248.826	534.503	191.458	725.961
**Met** ^**3**^	0	94.972	107.605	151.603	431.575	110.527	542.102	240.577	187.565	68.692	256.257	273.693	151.407	75.154	226.561
**Ile** ^**3**^	0	0	201.231	0	0	0	0	0	330.304	0	330.304	0	0	0	0
**Leu** ^**3**^	0	792.580	783.473	221.215	0	1270.930	1270.930	1242.176	1486.901	1141.124	2628.025	1295.509	1822.587	1050.825	2873.412
**Phe** ^**3**^	0	319.426	325.491	326.258	586.791	456.192	1042.983	538.699	659.497	462.148	1121.645	674.240	648.039	484.046	1132.085
**His** ^**3**^	0	184.623	604.147	592.122	486.849	225.905	712.754	1081.911	302.686	60.797	363.483	1170.781	236.211	309.935	546.146
**Lys** ^**3**^	0	392.204	368.867	103.645	1355.253	834.458	2189.711	237.900	1168.512	693.039	1861.551	301.897	1212.762	725.096	1937.858
**Asp** ^**4**^	0	25.692	42.094	41.356	156.505	36.537	193.042	451.747	199.156	111.345	310.501	514.480	138.848	96.877	235.725
**Ser** ^**4**^	0	0	0	24.198	0	0	0	222.233	0	0	0	212.067	0	0	0
**Glu** ^**4**^	0	0	0	77.682	260.957	73.925	334.882	623.413	597.372	237.544	834.916	691.035	487.047	251.237	738.284
**Pro** ^**4**^	0	1751.295	2306.616	0	21.802	0	21.802	55.303	49.514	0	49.514	152.670	31.927	0	31.927
**Gly** ^**4**^	0	65.190	87.162	31.687	164.689	37.076	201.765	299.986	164.738	119.370	284.108	350.020	165.579	120.686	286.265
**Ala** ^**4**^	0	0	0	40.558	1172.120	133.314	1305.434	474.570	626.602	229.531	856.133	531.357	579.769	243.989	823.758
**Cys** ^**4**^	0	0	0	0	333.820	0	333.820	0	0	0	0	0	0	0	0
**Tyr** ^**4**^	0	0	0	229.201	541.631	388.603	930.234	669.422	733.201	482.645	1215.846	624.271	738.280	485.299	1223.579
**Arg** ^**4**^	0	10.634	6.793	15.681	421.154	251.463	672.617	41.826	514.483	301.067	815.550	54.350	504.479	302.606	807.085
**Total**	**0**	**3636.616**	**4833.479**	**1938.677**	**7061.997**	**4448.483**	**11510.480**	**6794.662**	**9003.820**	**4548.052**	**13551.872**	**7521.277**	**8386.177**	**4735.373**	**13121.550**

1 Hydrolysis with pepsin, trypsin, chymotrypsin and carboxypeptidase-A

2 Total of released amino acids by dialyzability (Dext + Dint) NH = non-hydrolyzed; H1 = low degree of hydrolysis; H2 = high degree of hydrolysis; GD = gastric digest; Dext = external intestinal digest, samples collected inside of the membrane; Dint = internal intestinal digest, samples collected outside of the membrane

3 Essential amino acids

4 Non-essential amino acids

Another study [52] evaluated the antioxidant activity of free amino acids, showing *in vitro* that Tyr and Met have high antioxidant character. Thus, a higher antioxidant activity found in the sample H2Dint is possibly due to the presence of higher concentrations of Tyr, Phe, Leu and Val. In addition, HPLC analysis of the sample H2 showed the presence of several peaks which correspond to peptide bonds, releasing peptides (Fig 6C), which may also be contributing to the antioxidant activity obtained.

[53]. The antioxidant activity of whey proteins hydrolysates depends directly on the molar mass of the peptide, being the smallest ones (0.1–2.8 kDa) those with strong ability to scavenge free radicals.

### Amino acids released before and after gastric and intestinal enzymatic digestion

A greater variety of amino acids was obtained after gastric and intestinal enzymatic digestion when compared with NH, H1 and H2 samples (Table 2). M1 method released nine types of amino acids in the H1 sample, five of which are essential (methionine, leucine, phenylalanine, histidine and lysine), whereas ten types were detected in the H2 sample, six of which are essential (methionine, leucine, isoleucine, phenylalanine, histidine and lysine), as shown in Table 2. These results confirmed that amino acids and many peptides were released after enzymatic hydrolysis for H1 and H2 samples in relation to NH (Fig 6B and 6C).

Samples NH, H1 and H2 subjected to *in vitro* gastric and intestinal enzymatic digestion showed a 593.7% increase in the concentration of amino acids released for the NH sample when compared with gastric digestion only (pepsin action), whereas the increases for H1 and H2 samples were approximately 99% and 75%, respectively.

Under the study conditions and after a 2h dialyzability period, we demonstrated that diffusion through the semipermeable membrane was 38.6% for the NHDint, 33.6% for the H1Dint and 36.1% for the H2Dint.

This work demonstrated that the *in vitro* digestion of integral buffalo cheese whey proteins (NH) was effective in releasing at least 14 amino acids. Seven of the released amino acids are essential; the highest concentrations were of valine, leucine, phenylalanine and lysine, which were diffused through the membrane in the percentages 17.5%, 38.1%, 43.7% and 100%, respectively.

Tyrosine and arginine also showed significant diffusion rates (41.8% and 37.4%, respectively). The diffusion rates of the other amino acids were below 32%, showing that enzymatic digestion was effective in releasing essential amino acids and that buffalo cheese whey may become an important source of these compounds. Notably, the detection of amino acids by the method employed directly depends on their net charges, which change according to test conditions due to the different pH ranges employed.

Residues of isoleucine, tryptophan, asparagine and glutamine were not detected in any of the digestion steps.

Due to the prior hydrolysis of H1 and H2, a higher concentration of free amino acids was released and several peptides were formed. After the enzymatic digestion of both H1 and H2 samples, at least 14 of the twenty amino acids directly involved in bodily functions were released and detected after dialyzability; most of these amino acids are essential for mammals, including humans, and must be included in the diet. Among the essential amino acids, leucine was released in higher concentrations from H1 and H2, with diffusions of 43.4% and 36.6%, respectively. Leucine is involved in important processes, such as synthesizing and degrading muscle proteins and in stimulating the release of insulin from the pancreas. Thus, leucine is already being seen as a pharmaconutrient of great relevance for the supplementary feeding of malnourished and frail elderly and for specific subpopulations. In the case of the elderly, leucine could minimize sarcopenia, particularly in patients with type 2 diabetes due to its insulinotropic properties. In addition, leucine can also aid in muscle protein synthesis because there is an accelerated decline of muscle mass in these people [54–55]. Other amino acids, such as lysine, threonine, tyrosine and phenylalanine, were also released in high concentrations by the enzymatic digestion of the H1 and H2 hydrolysates. The percentage of diffusion in both cases was approximately 37% for lysine, 25 to 26% for threonine, 40% for tyrosine and 41 to 42% for phenylalanine, demonstrating that greater diffusion is not directly related to increased release because dialyzability is an *in vitro* method that provides us only a preliminary result that requires further *in vivo* studies. The remaining released amino acids, even at lower concentrations and diffusion percentages, are involved in regulating processes such as gene expression. *In vitro* studies indicate that dietary supplementation with glutamine and arginine increased the expression of genes with antioxidant properties, reducing the expression of pro-inflammatory genes. Another regulatory function attributed to amino acids is the synthesis and secretion of hormones, such as tyrosine and phenylalanine, which are precursors of the synthesis of epinephrine, norepinephrine, dopamine and thyroid hormones [56]. There is also a well-established immunological function related to the amino acids glutamine and arginine, methionine and cysteine, among others [56–57].

The hydrolysis of the proteins before gastrointestinal digestion favored the greater qualitative and quantitative release of amino acids. This finding was also observed in recent studies performed in our laboratory with bovine milk whey [26]. In relation to this same work, the amount of released amino acids from buffalo milk whey is much higher than the amount released from bovine milk whey. Our findings are interesting from a nutritional perspective because the use of whole proteins as a supplement produces the same effect as a pre-hydrolyzed supplement.

## Conclusions

In this study the protein profile of the buffalo cheese whey was analyzed in comparison with the bovine milk whey. The profiles are highly similar, except for one protein that is not present in the bovine milk whey. This protein, with 24 kDa, was analyzed by mass spectrometry, and the obtained peptides showed greater homology for β-lactoglobulin, being possibly a variant of this protein. Enzymatic hydrolysis, followed by *in vitro* gastrointestinal digestion, increased the release of amino acids, most of which essential amino acids, in higher concentrations when compared with bovine milk whey and able to scavenge free radicals. Buffalo cheese whey was found to be a successful alternative source of essential amino acids while presenting high biological value proteins. Moreover, these amino acids are bioavailable to perform their respective physiological functions.
